# Fine Structure of the Sensilla and Immunolocalisation of Odorant Binding Proteins in the Cerci of the Migratory Locust, *Locusta migratoria*

**DOI:** 10.1673/031.011.5001

**Published:** 2011-04-14

**Authors:** Yanxue Yu, Shuhui Zhou, Shangan Zhang, Long Zhang

**Affiliations:** ^1^ Institute of Animal and Plant Quarantine, Chinese Academy of Inspection and Quarantine, Beijing, China 100029; ^2^ Key Lab for Biocontrol of Pests, The Ministry of Agriculture, National Key Laboratory for Agricultural Biotechnology, China Agricultural University, Beijing, China 100193; ^3^ Shanghai Entry-Exit Inspection and Quarantine Bureau, Shanghai, China 200135

**Keywords:** cerci, chemoreception, scanning electron microscopy, Schistocerca gregaria, transmission electron microscopy

## Abstract

Using light and electron microscopy (both scanning and transmission), we observed the presence of sensilla chaetica and hairs on the cerci of the migratory locust, *Locusta migratoria* L. (Orthoptera: Acrididae). Based on their fine structures, three types of sensilla chaetica were identified: long, medium, and short. Males presented significantly more numbers of medium and short sensilla chaetica than females (p<0.05). The other hairs can also be distinguished as long and short. Sensilla chaetica were mainly located on the distal parts of the cerci, while hairs were mostly found on the proximal parts. Several dendritic branches, enveloped by a dendritic sheath, are present in the lymph cavity of the sensilla chaetica. Long, medium, and short sensilla chaetica contain five, four and three dendrites, respectively. In contrast, no dendritic structure was observed in the cavity of the hairs. By immunocytochemistry experiments only odorant-binding protein 2 from *L. migratoria* (*Lmig*OBP2) and chemosensory protein class I from the desert locust, *Schistocerca gregaria* Forsskål (*Sgre*CSPI) strongly stained the outer lymph of sensilla chaetica of the cerci. The other two types of hairs were never labeled. The results indicate that the cerci might be involved in contact chemoreception processes.

## Introduction

It is known that locusts perceive external stimuli through their chemosensory organs present on antennae, tarsi, wings, maxillary, and labial palps and cerci. Four types of chemosensilla are distributed on the antenna of the migratory locust, *Locusta migratoria* L. and the desert locust, *Schistocerca gregaria* Forsskål (Orthoptera: Acrididae), namely sensilla trichodea, s. chaetica, s. basiconica, and s. coeloconica (Ochieng et al. 1998; Jin et al. 2005). Some sensilla of the palps have been reported to play an important role in food selection (Blaney and Chapman 1969, 1970; Blaney et al. 1971; Klein 1981; Jin et al. 2006). Only one type of chemosensilla (sensilla chaetica) and three types of hairs were observed on the wings of *L. migratoria*, located on several veins on the forewing and hindwing of the locust (Zhou et al. 2008).

In contrast to detailed, knowledge of the anatomy, physiology, and role of sensilla on antennae, palps, and wings, little information is known about hairs on the cerci. Primitive hexapods have abdominal structures that represent modified remnants of ancestral walking limbs. Many hexapods have cerci (sensory appendages) on the 11^th^ abdominal segment. Cerci can be long with numerous segments or short with a single segment. Earwigs (*Labidura japonica*) have a pair of very long cerci at the posterior end of the abdomen that are larger and morphologically different in the male. It has been suggested that the cerci in this species may be used in defense, in catching insects and holding them while eating, in helping to fold the hindwings under the forewings. However, there is little information on the fine structure and distribution of chemosensilla on the cerci of earwigs. The cerci of the cockroach *Blattella* *germanica* have 10 segments with a number of sensilla chaetica B, speared sensilla, chaetica C and cone-shaped sensilla on the ventral surface, and a number of sensilla chaetica A, together with a few micro-trichoid sensilla on the back (Yang and Xu 1997). The cerci of *Periplaneta americana*, as observed by SEM, include pits and silk-like structures in addition to knobs between segments and sensilla chaetica A (Cheng et al. 1999).

Three OBPs and three CSPs were identified in locusts: OBP1 (Ban et al. 2003a), OBP2, and OBP3 (Yu et al. 2009) as well as CSPII (Ban et al. 2003b) from *L. migratoria* and CSPI (Angeli et al. 1999) and CSPIII (Jin et al. 2005) from *S. gregaria*. In this paper we report for the first time on the fine structure and classification of chemosensilla on the cerci of *L. migratoria*. Immunocytochemistry experiments revealed that sensilla chaetica is labeled by the antiserum *Lmig*OBP2 from *L. migratoria* and antiserum *Sgre*CSPI from *S. gregaria*, suggesting chemoreception functions of the cerci during the mating.

## Materials and Methods

### Insects

Adult *L. migratoria* in their gregarious phase were reared at the Department of Entomology, China Agricultural University (Beijing) at a temperature of 28–30° C, relative humidity of 60%, and a photoperiod of 18:6 L:D. Fresh wheat shoots were provided daily. The cerci from female and male adults were dissected for the experiments just after emergence.

### Light microscopy

For light microscopy (LM) the cerci were treated with 10% sodium hydroxide overnight and dehydrated by immersion in 100% ethanol, followed by 1:1 ethanol/xylene, and 100% xylene. The cerci were spread on a slide and mounted in Canadian gum. Five cerci were used for each sex.

### Scanning electron microscopy (SEM)

Cerci were excised and dried at room temperature for scanning electron microscopy. The samples were mounted on holders and examined by a HITACHI S570 (www.hitachihta.com) or FEI Quanta 200 (www.feicompany.com) SEM after coating in gold. As for light microscopy, five cerci were observed from each sex.

### Transmission electron microscopy (TEM)

For TEM, cerci were cut and fixed overnight with 2.5% glutaraldehyde in 0.1 M phosphate buffer solution, pH 7.4 (PBS), then rinsed for 30 min in PBS (4 changes), post-fixed with 2% OsO4 in 0.1 M PBS, and dehydrated in an ethanol series followed by 100% acetone. The fixed cerci were embedded in Epon 812 using propylene oxide. Ultra-thin sections were cut at the base of the sensilla with a glass knife on an LKB V Ultramicrotome and stained with uranyl acetate and lead citrate in an LKB ultrastainer before being mounted onto Formvar-coated grids. The specimens were observed using a HITACHI H-7500 TEM.

### Western blotting and Immunocytochemical localization

In order to test the specificity of antisera against chemosensory proteins and odorantbinding proteins identified in *L. migratoria* and *S. gregaria* (Angeli et al. 1999; Ban 2003a, 2003b; Yu et al. 2009), the four recombinant proteins *Lmig*CSPII, *Lmig*OBP1, *Lmig*OBP2, and *Lmig*OBP3 were electroblotted onto nitrocellulose (NC) membrane (Millipore HAHY00010, www.waters.com) and stained with the crude antiserum against recombinant SgreCSPI (Angeli et al. 1999). Immunoreacting bands were detected by treatment with 4-chloro-1-naphthol. Using the same method the recombinant proteins of *Lmig*CSPII and *Lmig*OBP2 with the antiserum against *Lmig*OBP2 was tested.

For immunocytochemistry, cerci were chemically fixed in a mixture of paraformaldehyde (4%) and glutaraldehyde (2%) in 0.1 M PBS (pH 7.4), dehydrated in an ethanol series and embedded in LR White resin (Taab, www.taab.co.uk) with polymerization at 60° C. Ultrathin sections (60–80 nm) were cut with a glass knife on a RMC MT-XL or with a diamond knife on a Reichert Ultracut ultramicrotome (www.reichert.com). The grids were subsequently floated on droplets of the following solutions: PBS containing 50 mM glycine, PBGT (PBS containing 0.2% gelatine, 1% bovine serum albumin and 0.02% Tween-20), primary antiserum diluted with PBGT, six washing with PBGT, secondary antibody diluted with PBGT, and two washings with PBGT, PBS glycine, PBS, and water. Silver intensification (Danscher 1981) was used to increase the size of the gold granules from 10 to about 40 nm, and treatment with 2% uranyl acetate to increase the contrast in transmission electron microscopy (HITACHIH-7500).

Three primary antisera were used at dilutions of 1:500, *Sgre*CSPI (CSPI of *Schistocerca gregaria*, Angeli et al. 1999; GenBank accession no. AF070964), *Lmig*OBP1 (OBP of *L. migratoria*, Ban et al. 2003a, GenBank accession no. AY542076), and *Lmig*OBP2 (OBP of *L. migratoria*, Yu et al. 2009, GenBank accession no. ACI30696) and incubated at 4° C overnight. As a control, the primary antiserum was replaced by serum from a healthy rabbit at the same dilution. None of the controls showed any labeling except few scattered grains in the background. The secondary antibody was anti-rabbit IgG, coupled to 10-nm colloidal gold (AuroProbe™ EM, GAR G10, Amersham, www.gelifesciences.com), diluted 1:20, and incubated at room temperature for 60–90 min.

**Table 1.**
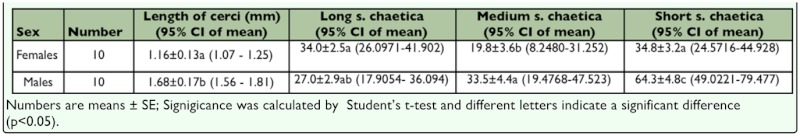
The number of chemosensilla of the cerci of *Locusta migratoria*

## Results

### Spatial map of the distribution of chemosensilla on the cerci

**Figure 1.**
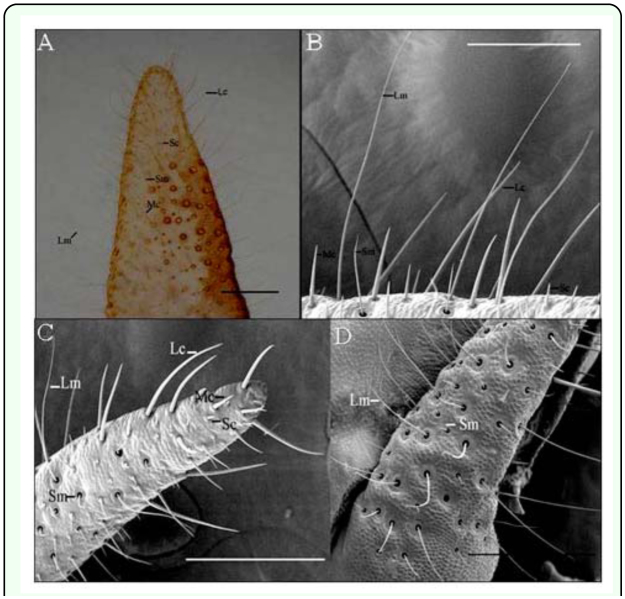
Spatial map and distribution of sensilla on the cerci of *Locusta migratoria*. Results from light microscopy (A) and scanning electron microscopy (B) show that there are three types of sensillum chaetica (long s. chaetica (Lc), medium s. chaetica (Mc), and short s. chaetica (Sc)) and two types of hairs (long hairs (Lm) and short hairs (Sm)). The distribution of the three types of s. chaetica and two types of hairs are shown in C and D. Bar: A = 180 µm; B = 200 µm; C, D = 400 µm. High quality figures are available online.

The cerci of *L. migratoria* are a pair of accessorial organs located on both sides of the 10^th^ segment of the abdomen. The cercus is cone-shaped and formed by a single segment with a sclerotic epidermis. Both light microscopy and scanning electron microscopy showed the presence of pegs and long hairs on the epidermis. Based on their external morphology and ultrastructure, five types of hairs were observed: long, medium, and short sensilla chaetica and long and short hairs (Figure 1). Long sensilla chaetica were mainly found on the tip of the cerci, while those of the medium type were fewer in number and principally located on the middle of the forepart of the cerci. Short sensilla chaetica were the most numerous and were evenly distributed across the forepart (Figure 1C). Few chemosensilla were found on the basal region of the cerci while long hairs, which were more numerous than their short counterparts, were evenly distributed (Figure 1D).

The numbers of long, medium, and short s. chaetica are reported in Table 1. The cercus of males were significantly longer than those of females, and the number of medium and short s. chaetica in males were higher than that in females (P<0.05). There was no difference in the number of long s. chaetica between the sexes (P<0.05).

### Long sensilla chaetica

**Figure 2.**
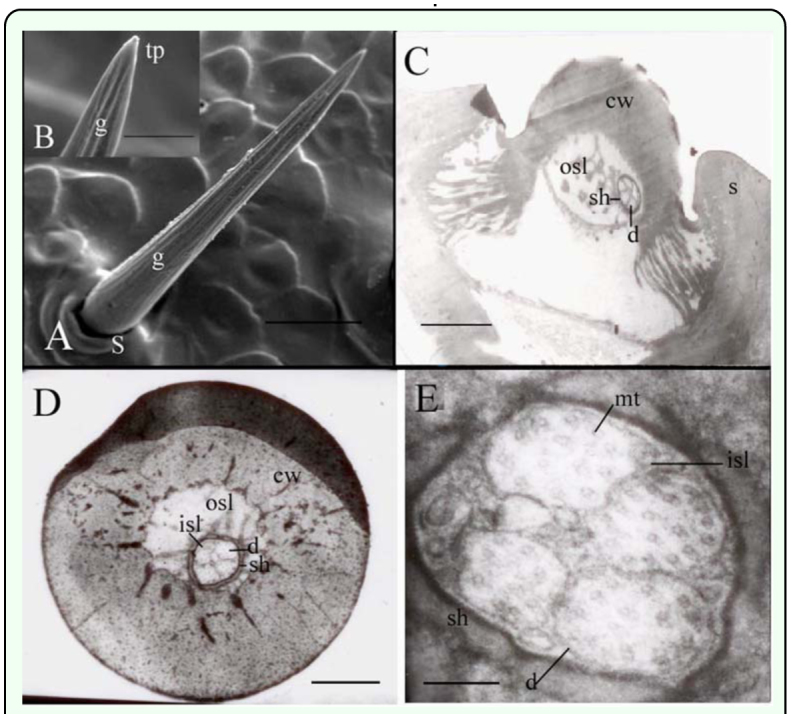
Fine structure of the long sensilla chaetica of the *Locusta migratoria* cerci. (A) The longitudinal grooves (g) on the surface are obvious together with a large socket (s) at the base but no pore on the cuticle wall (cw). (B) The groves are evident. (C) The longitudinal sections show the presence of five non-branched dendrites (d) in the inner sensillum lymph (isl). (D) The transverse sections show that the inner sensillum lymph (isl) and the outer sensillum lymph (osl) are separated by a dendritic sheath (sh). (E) Different dendrites vary in their number of microtubules (mt). Bar: A = 50 µm; B = 10 µm; C = 3 µm; D = I µm; E = 143 nm. High quality figures are available online.

Long s. chaetica were 180–300 µm in length with a diameter of around 13–20 µm at the base and 8–12 µm in the middle. They showed a slight curvature and longitudinal grooves on the surface, without pores on the surface (Figure 2). A single pore was observed at the tip of the peg (Figure 2B). They were 5–8 µm long and equipped with a socket at the base of around 24–41 µm in outer diameter, 15–21 µm in inner diameter.

The cuticular wall of long s. chaetica was around 6 µm thick and the inner diameter was about 8 µm (Figure 2D). The inner and the outer sensilla cavities were separated by a dendritic sheath. This class of s. chaetica contained five dendrites, enveloped by the dendritic sheath, in the inner lymph cavity that ran from the base to the tip (Fig. 2C, D, E). At its base, the dendritic sheath was 1.1–1.5 µm in diameter with the dendrites measuring 0.1– 0.2 µm in diameter. Four microtubules were present in slim dendrites, and 10–20 were present in thicker ones (Figure 2E).

### Medium sensilla chaetica

**Figure 3.**
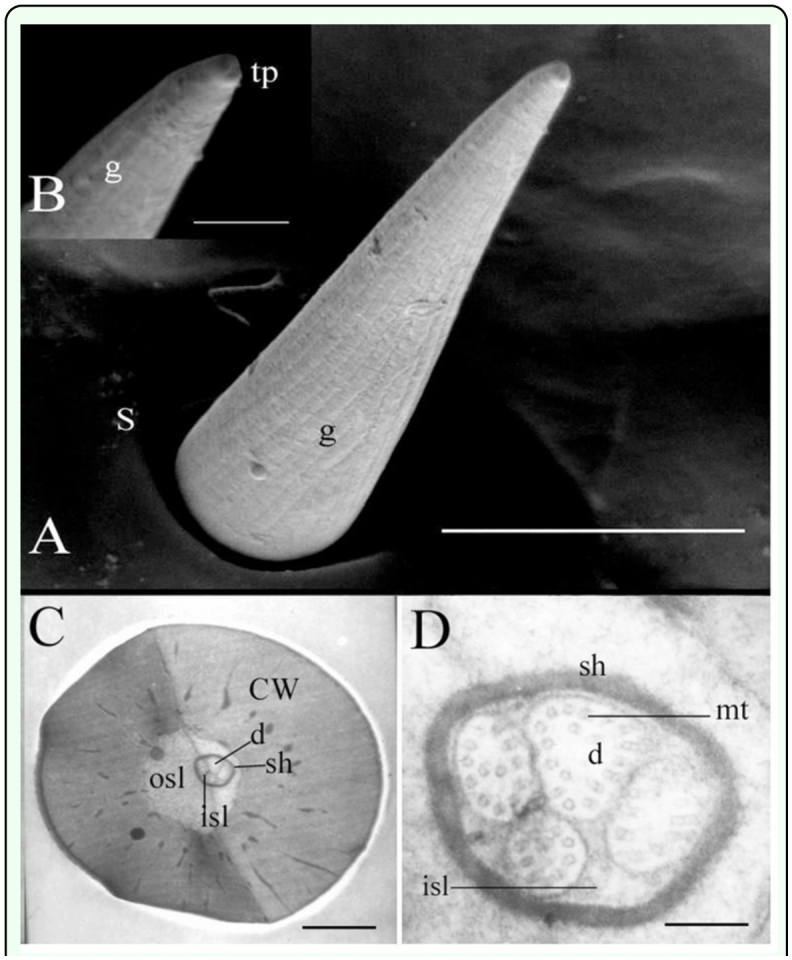
Fine structure of the medium sensillum chaetica of the *Locusta* *migratoria* cerci. (A) The longitudinal grooves (g) on the surface are obvious together with a large socket (s) at the base but no pore on the cuticle wall (cw). (B) The pore at the tip (tp). (C) The transverse sections show that the inner sensillum lymph (isl) contains four dendrites (d) enveloped by a dendritic sheath (sh). The outer sensillum lymph (osl) is also labeled. (D) The four dendrites vary in the number of microtubules (mt) from 10 to 20. Bar: A = 10 µm; B = 2 µm; C = I µm; D = 143 nm. High quality figures are available online.

Medium s. chaetica were 30–50 µm in length with a diameter at the base of around 8–13 µm and were equipped with a socket at the base of about 12–18 µm in outer diameter (Figure 3A). These s. chaetica stood erect, had one pore at the tip of the peg (Figure 3B) and longitudinal grooves on the surface (Figure 3A).

The cuticular wall was approximately 4–6 µm thick with an inner diameter of about 4 µm (Figure 3C). Four dendrites, with a diameter of 0.1–0.2 µm, were present within the inner lymph cavity (Figure 3C). The dendritic sheath was 1.5–2 µm in diameter at the base. The thinner dendrites contained 10 microtubules, the thicker ones around 20 (Figure 3D).

### Short sensilla chaetica

**Figure 4.**
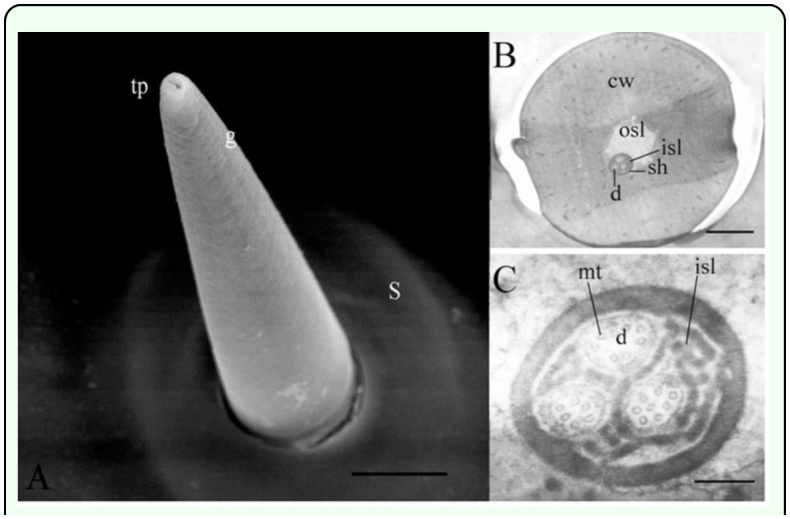
Fine structure of the short sensillum chaetica of the *Locusta migratoria* cerci. (A) The longitudinal grooves (g) on the surface are not visible together with a large socket (s) at the base. One pore is present on the tip (tp), but none is evident on the cuticle wall (cw). (B) The transverse section show that the lymph is separated by the dendritic sheath (sh) into the outer sensillum lymph (osl) and inner sensillum lymph (isl). Three nonbranched dendrites (d) are located in the inner sensillum lymph (isl); (C) The dendrites each have around 10 microtubules (mt). Bar: A = 5 µm; B = I µm; C = 125 nm. High quality figures are available online.

Short s. chaetica were 15–30 µm in length, had a diameter at the base of approximately 5–8 µm and a diameter at the tip of around 1.1–1.8 µm. A socket at the base of about 12 µm in outer diameter (Figure 4A) was evident. These s. chaetica also stood vertically and had one pore at the tip, but the longitudinal grooves on the surface were not evident (Figure 4A).

The cuticular wall of short s. chaetica was about 3 µm thick at the base. Three dendrites were present at the base of the sensilla (Figure 4B) with between 7 and 10 microtubules (Figure 4C).

### Long hairs

**Figure 5.**
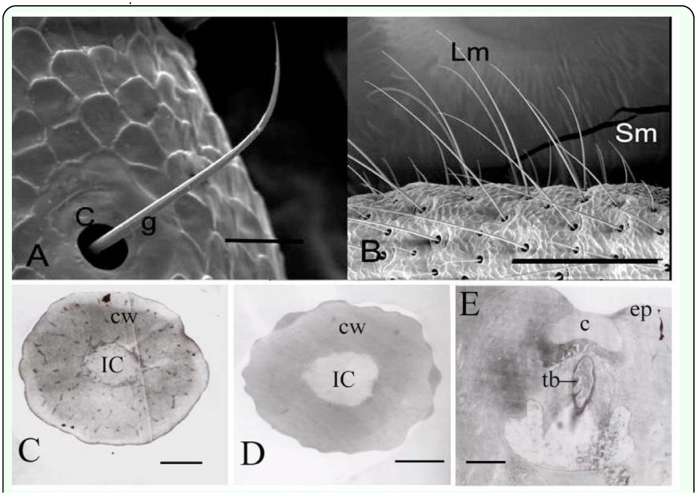
Fine structure of the hairs of the *Locusta migratoria* cerci. (A) Scanning electron microscopy shows that the long and short hairs have a similar morphology. Both are slight, have obvious longitudinal grooves (g) on the surface and a large cavity (c) at the base but no pore on the cuticle wall (cw). (B) Long and short hairs differ significantly in length. The transverse sections of long (C) and short hairs (D) indicate that there is only one inner cavity (IC) and no dendrites or dendritic sheath. (E) The longitudinal sections show tubular body (tb) and cavity (c). Epidermis is indicated by the abbreviation, ep.. Bar: A = 20 µm; B = 200 µm; C = 1.7 µm; D = 833 nm; E = 1.4 µm. High quality figures are available online.

The long hairs had a length of 250–400 µm, a base of about 4–7 µm in diameter and were evenly tapered from base to tip. A cavity at the base of approximately 10 µm diameters was apparent (Figure 5A). The hairs were curved and lacked pores on the wall, but possessed evident longitudinal grooves on the surface (Figure 5A, B).

The cuticular wall was about 3 µm thick at the base. A single inner cavity was observed, but there were no dendrites and dendritic sheath (Figure 5C, D). Longitudinal sections, however, revealed the presence of a tubular body (Figure 5E).

### Short hairs

The short hairs were 60–110 µm long and 3–5 µm wide at the base and, similarly to the long hairs, had a cavity at the base (Figure 5A, B). The inner structure was the same as in the long hairs.

### Western blotting and immunocytochemical localization

**Figure 6.**
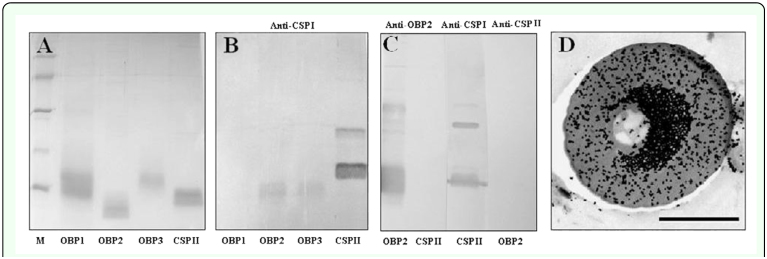
Western blot and immunocytochemistry. (A) The recombinant proteins of odorant-binding proteins (OBPs) and chemosensory proteins (CSPs): *Lmig*OBP1, *Lmig*OBP2, *Lmig*OBP3, and *Lmig*CSPII, were separated by 14% SDS-PAGE gels. Molecular weight markers are from the top: BSA (66kDa), ovalbumin (45kDa), carbonic anhydrase (31kDa), trypsin inhibitor (20kDa), and lactalbumin (14kDa). (B) Result of Western blot using the antiserum *Sgre*CSPI after SDS-PAGE together with A. Only *Lmig*OBP1 could not react with anti-*Sgre*CSPI, and other proteins all cross-react with anti-*Sgre*CSPI. (C) Result of Western blot using the antiserum *Lmig*OBP2, *Sgre*CSPI, and *Lmig*CSPII after the recombinant proteins of *Lmig*OBP2 and *Lmig*CSPII separating by SDS-PAGE. *Lmig*CSPII and *Lmig*OBP2 could not cross-react with each other, and *Sgre*CSPI and *Lmig*CSPII cross-react with each other. (D) Immunocytochemistry using the antiserum *Lmig*OBP2 indicated that *Lmig*OBP2 specifically expressed in the outer sensillum lymph of s. chaetica. The cuticle was not specifically labeled. Bar: D = 0.5µm. High quality figures are available online.

The recombinant proteins of chemosensory proteins and odorant-binding proteins isolated from *L. migritoria, Lmig*CSPII, *Lmig*OBP1, *Lmig*OBP2, and *Lmig*OBP3, were run on SDS-PAGE gel (Figure 6A), and electroblotted onto NC membrane. The antiserum against the *S. gregaria* chemosensory protein, *Sgre*CSPI showed some cross-reaction with the recombinant proteins *Lmig*CSPII, *Lmig*OBP2, and *Lmig*OBP3, but not with *Lmig*OBP1 (Figure 6B). Also, no cross-reaction was observed between *Lmig*CSPII and the antiserum against *Lmig*OBP2 (Figure 6C). In immunochemistry experiments, no sensilla were labeled by the antiserum against *Lmig*OBP1. The antiserum *Lmig*OBP2 labeled in the outer lymph of s. chaetica (Figure 6D). The cuticle was not specifically labeled. The results of consecutive sections labeled with antiserum *Lmig*OBP2 and *Sgre*CSPI also showed that CSPI and *Lmig*OBP2 were co-expressed in one sensilla.

## Discussion

Only one type of s. chaetica is present on the surface of the antennae, palps, and wings of locusts; although differences in neuronal composition may be present (Ochieng et al. 1998; Blaney and Chapman 1969; Jin et al. 2005, 2006). However, the s. chaetica was found to exhibit three different morphological forms, probably related to the fact that the cerci come into contact with stimuli not only from air, but also from ground during mating preparation and oviposition. The antiserum to the odorant-binding protein, *Lmig*OBP2, reacted with the s. chaetica while the antiserum to the odorant-binding protein *Lmig*OBP1 did not react, suggesting that *Lmig*OBP2 may be involved in mating. This is the first report that odorant-binding proteins in the locust are expressed in chemosensory organs other than the antenna. In addition, *Sgre*CSPI and *Lmig*OBP2 were expressed in the same sensillum, as are OS-E and OS-F of *Drosophila* (Hekmat-Scafe et al. 1997).

The three types of s. chaetica differ in their fine structure. Only one type of contact chemosensilla (s. chaetica) on the antenna has been found to have five dendrites (Ochieng et al. 1998; Angeli et al. 1999; Jin et al. 2005) while two types of s. chaetica located on the submaxilary palps have five and nine dendrites, respectively (Blaney and Chapman 1969; Blaney et al. 1971). Labial palps have a third type of s. chaetica containing six dendrites (Jin et al. 2006), while those of the wings have five dendrites (Zhou et al. 2008). This study found that the long, medium, and short s. chaetica possess five, four, and three dendrites, respectively. The number of dendrites may be related to the length of sensilla, and different length may play different function on the cerci.

The cereal filiform sensilla of the cricket *Gryllus bimaculatus* have been studied by electron microscopy and electrophysiology (Keil and Steinbrecht 1984; Shimozawa and Kanou 1984a). Two types of filiform hairs were found with different physiological functions. The larger type, with a length exceeding 500 µm, appears to be sensitive to low frequency stimuli while those shorter than 500 µm are insensitive to such stimulation (Shimozawa and Kanou 1984b). The two different lengths of hairs in the cerci of locusts may also play different roles. Future electrophysiological analyses may further explain the function of cerci.

Gustatory and olfactory reception can activate important behaviours in locusts and other insects such as mating, feeding, and oviposition (Chapman 2003). S. chaetica on the submaxillary palps with a pore at the tip are involved in stimulation resulting from contact with hydrophilic substances (Blaney 1974). However, s. basiconica and s. trichodea having pores on the cuticle wall can perceive olfactory stimuli such as aggregation and sex pheromones (Ochieng and Hansson 1999). The absence of olfactory sensilla on the cerci shows that the cerci are not involved in the transmission of olfactory stimuli. However, the presence of three types of s. chaetica and the expression of *Lmig*OBP2 and *Sgre*CSPI indicates that the cerci play a complicated gustatory and tactile function, such as detecting the volatiles of the ground to prepare for mating. Their specific functions need to be researched using electrophysiological methods.

## Abbreviations:

LmigCSPII,: L. migratoria chemosensory protein;
LmigOBP1, LmigOBP2, and LmigOBP3,: L. migratoria odorant-binding proteins;
SgreCSPI,: S. gregaria chemosensory protein
